# Gender Differences in Palliative Care Experience and Quality Perceptions Among Cancer Patients in Saudi Arabia: Implications for Nursing Practice

**DOI:** 10.3390/curroncol33040234

**Published:** 2026-04-21

**Authors:** Muteb Aljuhani, Hanadi Dakhilallah, Rayhanah R. Almutairi, Asrar S. Almutairi, Waleed M. Alshehri, Thurayya Eid, Abdulaziz M. Alodhailah

**Affiliations:** 1 Department of Community Health, Mental and Psychiatric Nursing, Imam Mohammad Ibn Saud Islamic University (IMSIU), Riyadh 11564, Saudi Arabia; 2 Nursing Administration and Education Department, College of Nursing, Imam Mohammad Ibn Saud Islamic University (IMSIU), Riyadh 11564, Saudi Arabia; 3 Community and Psychiatric Mental Health Nursing Department, College of Nursing, Princess Nourah bint Abdulrahman University, Riyadh 11671, Saudi Arabia; rralmutairi@pnu.edu.sa (R.R.A.); aasalmutairi@pnu.edu.sa (A.S.A.); 4 Department of Medical-Surgical Nursing, College of Nursing, King Saud University, Riyadh 11451, Saudi Arabia

**Keywords:** palliative care, gender differences, quality of care, cancer, nursing, Saudi Arabia, patient satisfaction

## Abstract

Gender shapes healthcare experiences, yet its influence on palliative care (PC) in Middle Eastern contexts remains under-researched. Understanding how male and female patients differently experience PC is essential for delivering gender-sensitive nursing care. A cross-sectional study was conducted among 200 cancer patients recruited from healthcare facilities in Saudi Arabia. PC quality perceptions were measured using the Palliative Care Experience and Quality Questionnaire (PCEQQ), assessing communication, symptom management, emotional support, and overall quality. Independent-samples *t*-tests compared mean scores between male (*n* = 99) and female (*n* = 101) participants. Chi-square tests and logistic regression examined gender differences in access and associated factors. Female patients reported significantly higher overall satisfaction (M = 4.12 vs. M = 3.85, *p* = 0.008) and communication quality (M = 4.10 vs. M = 3.65, *p* = 0.002) compared to males. Females also demonstrated greater PC awareness (69.3% vs. 47.5%, *p* = 0.008) and access rates (64.4% vs. 47.5%, *p* = 0.013). Emotional support ratings approached significance, favoring females (*p* = 0.052), while symptom management showed no significant gender differences. These findings suggest that gender significantly shapes the PC experience in Saudi Arabia, with the most robust disparity appearing in provider, patient communication. Nurses should adopt gender-sensitive strategies to bridge the engagement gap for male patients, who may face cultural barriers to accessing supportive care.

## Simple Summary

This study aims to understand how gender differences influence the awareness and experiences of cancer patients regarding palliative care in Saudi Arabia. We found that female patients tend to have better knowledge of these services, use them more frequently, and feel more satisfied with the quality of care compared to male patients. The most striking difference was observed in how healthcare providers communicate, with male patients reporting lower satisfaction than females. This may be influenced by cultural norms where men feel pressure to remain stoic or are less comfortable discussing emotional vulnerability. These findings are vital for nurses to adopt gender-sensitive approaches, ensuring male patients feel more supported and comfortable communicating their needs. By recognizing these unique requirements, healthcare services can provide more equitable and high-quality care for all oncology patients regardless of their gender.

## 1. Introduction

Gender constitutes a fundamental determinant of health behaviors, healthcare utilization, and care experiences across the lifespan [1]. Internationally, women consistently demonstrate more proactive engagement with healthcare services, including preventive care, supportive services, and patient education programs [2]. These patterns reflect complex interactions between biological, psychological, and socio-cultural factors that shape health-seeking behaviors differently for men and women [3,4].

In palliative care (PC), gender-sensitive approaches are increasingly recognized as essential for equitable service delivery [5,6]. PC addresses multidimensional suffering encompassing physical, psychological, social, and spiritual domains, which are all areas where gender may influence patient needs, preferences, and responses to care [7,8]. Yet research examining gender differences in PC experience and quality perceptions remains limited, particularly in Middle Eastern contexts where cultural norms and gender roles differ substantially from Western settings. A recent scoping review confirmed that gender shapes multiple dimensions of end-of-life care, including symptom experience, care preferences, and coping, yet quantitative evidence from non-Western settings remains sparse [9]. Foundational calls to integrate gender into palliative research and policy have highlighted its systematic neglect [10], and systematic reviews document that age- and gender-based inequalities in palliative care access persist across cancer populations internationally [11].

Saudi Arabia presents a unique context for examining gender and PC. Rapid social transformations within the Vision 2030 framework have enhanced women’s participation in public life, yet traditional gender roles persist within families and healthcare settings [12,13,14,15]. Women frequently serve as primary caregivers for ill family members, potentially increasing their familiarity with supportive care services [16,17]. Conversely, cultural norms emphasizing masculine strength may discourage men from acknowledging vulnerability or seeking emotional support [18,19]. Understanding how these dynamics influence PC access and experience is essential for developing gender-sensitive nursing interventions.

Nurses are ideally positioned to address gender-based disparities in PC through their central roles in patient education, communication, and psychosocial support [20,21]. By recognizing how gender shapes care experiences, nurses can adapt communication strategies, educational approaches, and support interventions to meet the distinct needs of male and female patients. Such tailored approaches may be particularly important in contexts where cultural factors amplify gender differences in healthcare behavior.

This study aimed to examine gender differences in PC access, awareness, and quality perceptions among cancer patients in Saudi Arabia. We hypothesized that female patients demonstrate greater awareness, higher access rates, and more favorable quality perceptions compared with males. The findings carry implications for nursing practice in delivering gender-sensitive PC to diverse patient populations.

## 2. Materials and Methods

The study was conducted across multiple healthcare settings in Riyadh, Saudi Arabia, including tertiary oncology centers, regional hospitals, and patient support organizations, ensuring diverse representation of patients from various socio-economic backgrounds. Data collection took place over a few-month period from November to March 2025.

Adult cancer patients (≥18 years) receiving active oncology treatment or follow-up care were eligible for participation. Inclusion criteria encompassed a confirmed cancer diagnosis, Saudi Arabian residency, and the capacity to provide informed consent. Exclusion criteria included severe cognitive impairment or illness severity precluding participation. Purposive sampling supplemented by convenience recruitment yielded 200 participants (99 males, 101 females), providing adequate power for between-group comparisons. Research nurses approached consecutive eligible patients during routine clinic visits over a 6-month recruitment period.

### 2.1. Instruments

Three instruments were used for data collection. Socio-demographic and Clinical Information Form: Demographic variables (age, gender, marital status, education, income, and region) and clinical characteristics (cancer type, stage, treatment status, months since diagnosis) were captured using an investigator-developed tool with established content validity.

Palliative Care Awareness and Accessibility Scale (PCAAS): This 12-item instrument, adapted from the Palliative Care Knowledge Scale [22], measures awareness (7 items) and accessibility (5 items) on 5-point Likert scales (1 = strongly disagree to 5 = strongly agree). Items assess knowledge of PC concepts, eligibility criteria, and availability of services. Cronbach’s α = 0.84 in this sample. For analysis, awareness was dichotomized as high awareness (mean score ≥ 4.0) versus low awareness (<4.0).

Palliative Care Experience and Quality Questionnaire (PCEQQ): Adapted from the FAMCARE Patient Scale [23], this 20-item tool measures satisfaction across four domains: communication (5 items assessing clarity of information, listening, and understanding), symptom management (5 items addressing pain control, dyspnea relief, nausea management, constipation, and fatigue relief), emotional support (5 items covering emotional comfort, discussion of fears/concerns, spiritual support, family support, and dignity), and overall quality (5 items assessing global satisfaction, quality of life contributions, holistic care, continuity, and provider attentiveness). Items use 5-point Likert scaling (1 = very dissatisfied to 5 = very satisfied). Domain scores were calculated as mean item responses. Cronbach’s α = 0.91 overall in this sample (communication α = 0.88; symptom management α = 0.85; emotional support α = 0.84; overall quality α = 0.86).

### 2.2. Data Collection

Following ethical approval and informed consent, participants completed questionnaires on paper or in an electronic format during the data collection period from November to March 2025. Trained oncology nurses facilitated distribution and provided clarification as needed. Participants were assured that survey responses would not affect their medical care. Completion time averaged 10–15 min. Data were de-identified at entry and stored in encrypted, password-protected electronic databases.

Descriptive statistics summarized sample characteristics by gender. Chi-square tests examined gender differences in categorical variables (awareness levels, access rates). Independent-samples *t*-tests compared mean scores on PCEQQ domains between male and female participants, with 95% confidence intervals (CIs) calculated around mean differences. Effect sizes (Cohen’s d) were calculated for all *t*-test comparisons with the following interpretations: d < 0.20 (negligible), 0.20–0.50 (small), 0.50–0.80 (medium), and >0.80 (large). Logistic regression assessed gender as a predictor of access while controlling for age and income. Analyses were conducted in using IBM SPSS Statistics version 26.0 (IBM Corp., Armonk, NY, USA) with α = 0.05 for two-tailed significance testing.

Ethical approval was obtained from the Institutional Review Board of King Saud University (approval code: KSU-HE-24960; approval date: 5 November 2024). All study procedures were conducted in accordance with the Declaration of Helsinki principles and Saudi Arabia’s national research ethics guidelines. All participants provided written informed consent. Anonymity and confidentiality were maintained throughout data collection and storage. Participants were assured that incomplete responses or non-participation would not affect their medical care. Confidentiality was ensured through use of anonymous participant codes unrelated to identifying information; all data were stored on encrypted, password-protected servers with access restricted to the research team. Risks to participants were minimal; no adverse events or complaints were reported. Participants retained the right to withdraw from data collection at any time, though no withdrawals occurred.

## 3. Results

Table 1 presents sample characteristics stratified by gender. The 99 male and 101 female participants were comparable in age distribution, with mean ages of 47.8 years (SD = 10.2) and 48.7 years (SD = 9.1), respectively (t = −0.73, *p* = 0.465). Educational attainment was similar across genders, with approximately half of both groups (males 47.5%, females 49.5%) holding university degrees (χ^2^ = 0.08, *p* = 0.773). Income distribution was also comparable, with no significant gender differences in socio-economic status (χ^2^ = 0.20, *p* = 0.656). Marital status was equivalent across genders (males married 64.6%, females 61.4%; χ^2^ = 0.29, *p* = 0.591).

Clinical characteristics were similarly distributed: most participants (71%) were receiving follow-up care rather than active treatment (29%); median time since diagnosis was 2.3 years (IQR = 1.1–4.8); and breast cancer (38%), hematologic malignancies (22%), gastrointestinal cancers (21%), and urologic/other cancers (19%) comprised the cancer types. No significant gender differences emerged in cancer type distribution (χ^2^ = 1.94, *p* = 0.586) or disease stage (stage I–II: 45% vs. stage III–IV: 55%; χ^2^ = 0.12, *p* = 0.734).

Female patients demonstrated significantly higher awareness of PC compared with males. Among females, 69.3% (*n* = 70) reported high awareness (PCAAS awareness subscale mean ≥ 4.0) versus 47.5% (*n* = 47) of males; χ^2^ = 7.23, *p* = 0.008. This represented a difference of 21.8 percentage points in high awareness rates between genders. The odds of reporting high awareness were 2.48 times greater for females compared with males (OR = 2.48, 95% CI: 1.29–4.76, *p* = 0.006).

Table 2 presents item-level analysis of PC awareness. At the item level, females reported greater agreement that “palliative care is appropriate early in cancer treatment” (female M = 4.35, SD = 0.71 vs. male M = 3.87, SD = 0.89; t = −3.62, *p* < 0.001, d = 0.51) and that “I understand what palliative care means” (female M = 4.28, SD = 0.68 vs. male M = 3.95, SD = 0.84; t = −2.94, *p* = 0.004, d = 0.42). These findings suggest that female patients possessed more accurate and comprehensive knowledge of PC concepts, eligibility criteria, and appropriate timing of initiation.

Table 3 presents PC access rates by gender. Female patients were significantly more likely to have accessed PC services (64.4%, *n* = 65) compared with males (47.5%, *n* = 47); χ^2^ = 6.21, *p* = 0.013. The unadjusted odds ratio was 1.46 (95% CI: 1.09–2.31, *p* = 0.013), indicating that females had 46% greater odds of accessing PC compared to males. This gender difference persisted after controlling for age and income in logistic regression: adjusted OR = 1.52 (95% CI: 1.10–2.42, *p* = 0.009), suggesting that sociodemographic confounding did not substantially attenuate the association.

Table 4 presents independent-samples *t*-test results comparing quality perceptions between genders. Female patients reported significantly higher satisfaction across multiple PC dimensions. Overall quality ratings were significantly higher among females (M = 4.12, SD = 0.68) than males (M = 3.85, SD = 0.75); t(198) = −2.68, *p* = 0.008, Cohen’s d = 0.38 (small-to-medium effect). The 0.27-point difference on the 5-point scale represented a 5.4% absolute difference in satisfaction ratings.

The largest gender difference emerged in communication satisfaction, where females (M = 4.10, SD = 0.72) significantly outscored males (M = 3.65, SD = 0.80); t(198) = −3.10, *p* = 0.002, Cohen’s d = 0.59 (medium effect). This 0.45-point difference represented a 12% absolute difference in communication satisfaction and was the most robust gender disparity observed.

Symptom management ratings were slightly higher among females (M = 4.05, SD = 0.65) than males (M = 3.90, SD = 0.70), though this difference did not achieve statistical significance; t(198) = −1.50, *p* = 0.135, Cohen’s d = 0.22 (small effect). The 0.15-point difference was negligible in practical magnitude, suggesting that clinical aspects of PC are delivered relatively equitably across genders.

Emotional support ratings showed a pattern favoring females (M = 3.95, SD = 0.78) compared with males (M = 3.70, SD = 0.82); t(198) = −1.95, *p* = 0.052, Cohen’s d = 0.31 (small effect). While this difference approached but did not meet the conventional significance threshold (*p* = 0.052), the effect size was small to moderate, suggesting potential clinical relevance.

Table 5 presents item-by-item analysis of PCEQQ responses, revealing where gender differences in satisfaction emerge most prominently. Among communication items, all five showed significant gender differences, with females consistently rating provider communication higher than males. For symptom management, while the overall dimension was non-significant, individual items showed trends favoring females that did not reach significance. In the emotional support domain, specific items regarding emotional support availability and family support showed individual-level significance.

To examine whether cancer disease stage confounded observed gender differences in quality perceptions, stratified analyses were conducted comparing gender effects separately for early-stage (I–II) and advanced-stage (III–IV) disease. Table 6 presents these results. Gender differences in communication satisfaction remained significant across both stage groups: early-stage cancer (*n* = 90, females M = 4.15 vs. males M = 3.68, d = 0.62, *p* = 0.009) and advanced-stage disease (*n* = 110, females M = 4.07 vs. males M = 3.62, d = 0.57, *p* = 0.008). Similarly, overall quality satisfaction differences persisted across both stage subgroups (early-stage d = 0.41, *p* = 0.029; advanced-stage d = 0.36, *p* = 0.041), suggesting that disease severity did not substantially confound the observed gender associations.

Table 7 presents the logistic regression model predicting PC access with gender, age, and income as predictor variables. The unadjusted model showed that female gender was a significant predictor of access (OR = 1.46, *p* = 0.013). When age and income were added to the model, the adjusted odds ratio for female gender increased slightly to 1.52 (*p* = 0.009), indicating that the gender effect was not substantially confounded by these demographic variables. Age was not a significant predictor (OR = 0.98 per year, *p* = 0.612), and income ≥ 10,001 SAR showed a trend toward increased access (OR = 1.28, *p* = 0.089) but did not reach conventional significance.

Table 8 provides a summary of all key statistical findings from the study, organized by outcome domain (awareness, access, and quality perceptions) with effect sizes for easy reference and interpretation.

## 4. Discussion

This study provides novel evidence on gender differences in PC awareness, access, and quality perceptions among cancer patients in Saudi Arabia. Female patients consistently demonstrated greater engagement with PC services and more favorable quality assessments, particularly regarding communication, a finding with important implications for nursing practice.

The higher awareness among female patients aligns with the international literature documenting women’s greater health information-seeking behaviors [1]. In Saudi Arabia, women’s traditional caregiving responsibilities may enhance exposure to health services and information [16,24]. Female patients may also benefit from stronger social networks that facilitate health information exchange [25]. These awareness advantages translate into higher PC access rates, reinforcing the pathway from knowledge to utilization identified in prior research. It must be explicitly stated, however, that the cross-sectional design of this study precludes any causal interpretation of these associations. The observed relationship between gender and perceived quality of care reflects a statistical association only; we cannot determine whether gender itself produces these differences, or whether gender acts as a proxy for unmeasured variables such as social support availability, prior healthcare experiences, or cultural expectations of care. Readers and clinicians should interpret the directional language used throughout this discussion (e.g., “female patients demonstrated greater awareness”) as descriptive of observed patterns rather than as evidence of causal directionality. This limitation is especially salient when interpreting communication satisfaction differences, where bidirectional influences between patient behavior and provider behavior cannot be disentangled from cross-sectional data alone.

The substantial gender gap in communication satisfaction (Cohen’s d = 0.59) warrants particular attention. Male patients’ lower satisfaction with provider communication may reflect multiple factors. First, cultural norms emphasizing masculine stoicism may inhibit men from fully engaging in discussions about symptom burden, emotional distress, or end-of-life concerns [18]. Second, healthcare providers may inadvertently communicate differently with male patients, offering less emotional support or assuming men prefer directive rather than collaborative communication styles. Third, men may have different communication preferences that current PC delivery models inadequately address.

The trend toward lower emotional support satisfaction among males (*p* = 0.052) further suggests that PC services may be suboptimally meeting men’s psychosocial needs. While this difference did not achieve conventional statistical significance, the moderate effect size (d = 0.31) suggests clinical relevance. Male cancer patients may experience unmet emotional needs due to reluctance to express vulnerability or discomfort with traditional supportive care formats. Alternative approaches such as peer support groups, activity-based interventions, or technology-mediated support may better engage male patients [26].

The finding that symptom management ratings did not differ significantly by gender suggests that clinical aspects of PC may be delivered more equitably than psychosocial components. This is encouraging but also highlights that gender disparities concentrate in communication and emotional support domains, precisely those areas where nurses play central roles. Nursing interventions targeting these domains may be particularly effective in reducing gender inequities. An important caveat to the symptom management finding, and to the quality perception findings more broadly, is that this study did not collect data on baseline symptom severity, functional status (e.g., ECOG or Karnofsky performance scores), or comorbidity burden. These factors are well-established determinants of how patients perceive the adequacy of symptom control and overall care quality. It is possible that gender differences in perceived care quality are partly mediated by differences in symptom burden at baseline: if male patients entered the study with more severe or undertreated symptoms, their lower satisfaction scores might reflect objective differences in clinical status rather than differential treatment by providers. Conversely, if female patients had better functional status at enrollment, this could inflate their satisfaction ratings independently of care quality. Future studies should routinely incorporate validated functional status measures (e.g., the ECOG Performance Status Scale or the Edmonton Symptom Assessment System) to permit adjustment for these potential confounders and to clarify whether gender differences in satisfaction persist after accounting for clinical severity.

These findings support theoretical frameworks emphasizing gender as a social determinant of health behavior and healthcare experience [2,27]. The health belief model suggests that perceived susceptibility, severity, and benefits influence care-seeking: perceptions that may be shaped by gender-specific socialization [28,29]. Men who socialize to minimize illness and maintain self-reliance may perceive a lower need for supportive care and fewer benefits from help-seeking. Nursing interventions should address these gendered beliefs explicitly, normalizing PC engagement for male patients and emphasizing concrete benefits.

Social support theory also illuminates gender differences in PC experience [25]. Women’s typically larger and more emotionally intimate social networks may facilitate both information acquisition and emotional processing of illness. Male patients with smaller networks may rely more heavily on healthcare providers for support, yet simultaneously be less comfortable seeking such support. Nurses can help bridge this gap by proactively offering emotional support to male patients rather than waiting for explicit requests. Evidence from Saudi cancer care settings confirms that nurse–patient communication quality, emotional responsiveness, and information provision are all pivotal determinants of patient satisfaction [30,31]. Gender-specific needs also extend to family caregivers, where male and female caregivers report differing support requirements during specialist palliative care, further underscoring the importance of gender-sensitive team approaches [32].

These findings carry several implications for delivering gender-sensitive PC. First, nurses should recognize that male patients may face cultural barriers to engaging with supportive services and expressing emotional needs. Proactive outreach, normalization of PC, and validation of men’s experiences may enhance engagement. Assessment of psychosocial needs should not rely solely on patient-initiated disclosure, as men may underreport distress [18].

Second, communication strategies may require adaptation for male patients. Some men may prefer concrete, problem-focused communication over open-ended emotional exploration. Providing information about symptom management, prognosis, and practical planning may serve as entry points for broader supportive conversations. Incorporating male patients’ preferences for communication style and setting (e.g., during activities rather than formal counseling sessions) may enhance engagement.

Third, nurses should consider structural innovations to better engage male patients in PC. Technology-based interventions, peer support from male cancer survivors, and integration of PC education into routine oncology encounters may reduce barriers [33,34]. Family-inclusive approaches engaging spouses or partners may also facilitate male patients’ access to emotional support through trusted relationships.

Fourth, nursing education should incorporate content on gender-sensitive care delivery, preparing nurses to recognize and address gender-based differences in healthcare behavior and preferences. Continuing education programs can equip practicing nurses with strategies for engaging diverse patient populations, including men who may be reluctant to accept supportive services.

Fifth, these findings must be interpreted with careful attention to their geographic and institutional boundaries. All participants were recruited from tertiary oncology centers in Riyadh, an urban setting with comparatively high healthcare resource density and patient education levels. This sampling strategy likely selects patients with better access to information, stronger health literacy, and greater familiarity with formal healthcare structures than would be typical of Saudi Arabia’s rural or semi-urban populations. In rural regions, PC infrastructure is considerably less developed, healthcare provider density is lower, and cultural attitudes toward palliative and supportive care may differ substantially from those in Riyadh. The gender differences observed here—particularly in awareness and access—may therefore be attenuated or amplified in rural contexts, where barriers to PC access are both more severe and differently distributed between men and women. Generalization of these findings to rural Saudi Arabian populations, or to primary care settings, should be approached cautiously. Future research should deliberately recruit from rural governorates and primary healthcare centers to characterize whether the gender disparities documented here persist across the full geographic and socioeconomic range of Saudi Arabia.

## 5. Strengths and Limitations

This study possesses several methodological strengths. The balanced gender distribution (50.5% female) and comparable sociodemographic characteristics across genders reduce confounding by age, education, or socioeconomic status. Validated measurement instruments with strong internal consistency (PCEQQ α = 0.91; PCAAS α = 0.84) provide reliable measurement of PC quality and awareness. The comprehensive assessment of PC quality across four distinct dimensions moves beyond a single-outcome focus to illuminate where gender disparities emerge and where equitable delivery occurs. The Saudi Arabian setting addresses a critical research gap by providing the first quantitative evidence of gender differences in PC experiences in a Middle Eastern context, with direct relevance to nursing practice in culturally diverse settings. The reporting of effect sizes (Cohen’s d) alongside *p*-values facilitates clinical interpretation and meta-analytic synthesis. Stratified analyses by disease stage and examination of associations across cancer types demonstrate attention to potential confounding.

This study has several important limitations that constrain interpretation and generalizability. The cross-sectional design precludes causal inference regarding the direction of relationships between gender, awareness, and quality perceptions. Self-selection bias may have influenced participation, with more engaged patients potentially overrepresented. The convenience sampling limits generalizability to broader Saudi Arabian or regional populations. Quality perceptions were based on patient self-report rather than objective quality indicators. Disease severity indicators such as baseline symptom burden, functional status, and comorbidity count were not measured, potentially creating unmeasured confounding. Finally, the study was conducted in urban Riyadh at tertiary care settings; findings may not generalize to rural regions or primary care contexts. Future research should employ longitudinal designs, include qualitative exploration of gendered experiences, and evaluate interventions designed to enhance male patient engagement with PC services.

## 6. Conclusions

This study demonstrates significant gender differences in PC awareness, access, and quality perceptions among Saudi Arabian cancer patients, with female patients showing greater engagement and satisfaction across multiple dimensions. The substantial communication satisfaction gap between genders highlights an area requiring nursing attention. Gender-sensitive approaches to PC delivery, including proactive outreach to male patients, adapted communication strategies, and alternative support modalities, may reduce disparities and ensure equitable access to high-quality supportive care. Nurses occupy a central position in implementing such approaches through education, assessment, and individualized care planning. Regarding communication specifically, we recommend that nurses serve as the primary communicators for psychosocial and supportive care discussions with male patients, given their continuous bedside presence and established therapeutic relationships. However, a multidisciplinary approach is most effective: physicians should lead prognostic and treatment-related conversations, while social workers address practical and family support needs. In contexts where male patients exhibit reluctance to disclose emotional concerns, a coordinated team approach—with nurses initiating contact and facilitating referral to social workers or counselors—is likely to yield the most equitable outcomes.

## Figures and Tables

**Table 1:** Sample characteristics by gender.

Variable	Male (*n* = 99)	Female (*n* = 101)	Test Statistic	*p*-Value
Age, Mean (SD) years	47.8 (10.2)	48.7 (9.1)	t = −0.73	0.465
Education: University degree, *n* (%)	47 (47.5)	50 (49.5)	χ^2^ = 0.08	0.773
Monthly Household Income ≥ 10,001 SAR, *n* (%)	32 (32.3)	35 (34.7)	χ^2^ = 0.20	0.656
Marital Status: Married, *n* (%)	64 (64.6)	62 (61.4)	χ^2^ = 0.29	0.591
Treatment Status: Active treatment, *n* (%)	28 (28.3)	30 (29.7)	χ^2^ = 0.15	0.694
Months Since Diagnosis, Median (IQR)	2.2 (1.0–4.6)	2.4 (1.2–5.1)	-	0.381
Cancer Type, *n* (%)			χ^2^ = 1.94	0.586
Breast	35 (35.4)	41 (40.6)		
Hematologic	24 (24.2)	20 (19.8)		
Gastrointestinal	23 (23.2)	19 (18.8)		
Urologic/Other	17 (17.2)	21 (20.8)		
Disease Stage, *n* (%)			χ^2^ = 0.12	0.734
Stage I–II (Early)	45 (45.5)	45 (44.6)		
Stage III–IV (Advanced)	54 (54.5)	56 (55.4)		

Note: SAR = Saudi Arabian Riyal; IQR = interquartile range. No significant differences were detected between genders on any demographic or clinical variable, confirming demographic comparability across groups.

**Table 2:** Item-level analysis of palliative care awareness by gender.

PCAAS Item	Male Mean (SD)	Female Mean (SD)	*t*-Value	*p*-Value	Cohen’s d	Effect Size
Awareness Subscale Items						
1. I understand what palliative care means	3.95 (0.84)	4.28 (0.68)	−2.94	0.004 *	0.42	Small
2. PC is for end-of-life care only (reversed)	2.45 (1.12)	2.18 (1.08)	1.65	0.102	0.24	Negligible
3. PC is appropriate early in cancer treatment	3.87 (0.89)	4.35 (0.71)	−3.62	<0.001 *	0.51	Medium
4. PC focuses on comfort and quality of life	4.12 (0.79)	4.41 (0.60)	−2.58	0.011 *	0.38	Small
5. I know when PC is appropriate	3.68 (0.95)	4.09 (0.81)	−2.88	0.005 *	0.42	Small
6. PC includes emotional/spiritual support	3.92 (0.86)	4.25 (0.75)	−2.52	0.012 *	0.37	Small
7. PC improves quality of life	4.05 (0.80)	4.38 (0.62)	−2.89	0.004 *	0.43	Small
Awareness Subscale Mean	3.71 (0.68)	4.13 (0.55)	−4.12	<0.001 *	0.61	Medium
Accessibility Subscale Items						
8. PC services are available in my community	3.28 (1.08)	3.85 (0.94)	−3.58	<0.001 *	0.53	Medium
9. I know where to access PC services	3.15 (1.15)	3.74 (1.05)	−3.37	0.001 *	0.50	Medium
10. Healthcare providers recommend PC early	3.42 (1.02)	3.91 (0.88)	−3.32	0.001 *	0.49	Medium
11. Accessing PC is easy in my hospital	3.35 (1.10)	3.82 (1.00)	−2.99	0.003 *	0.44	Small
12. Information about PC is readily available	3.18 (1.12)	3.68 (1.08)	−2.95	0.004 *	0.44	Small
Accessibility Subscale Mean	3.28 (0.85)	3.80 (0.76)	−4.23	<0.001 *	0.63	Medium
PCAAS Total Mean	3.50 (0.67)	3.96 (0.59)	−4.83	<0.001 *	0.72	Medium

Note: PCAAS = Palliative Care Awareness and Accessibility Scale. All items were measured on a 5-point Likert scale (1 = strongly disagree to 5 = strongly agree). * = *p* < 0.05 (statistically significant). Cohen’s d interpretation: <0.20 (negligible), 0.20–0.50 (small), 0.50–0.80 (medium), >0.80 (large).

**Table 3:** Palliative care access by gender.

Variable	Accessed PC *n* (%)	Not Accessed PC *n* (%)	Total *n*	Unadjusted OR (95% CI)	*p*-Value (Unadjusted)	Adjusted OR (95% CI)	*p*-Value (Adjusted)
Male	47 (47.5)	52 (52.5)	99	Reference	-	Reference	-
Female	65 (64.4)	36 (35.6)	101	1.46 (1.09–2.31)	0.013 *	1.52 (1.10–2.42)	0.009 *
Overall	112 (56.0)	88 (44.0)	200	-	-	-	-

Note: OR = odds ratio; CI = confidence interval. Adjusted odds ratio controlled for age (continuous) and monthly household income (≥10,001 SAR vs. <10,001 SAR). * = *p* < 0.05 (statistically significant). Gender difference in PC access: 16.9 percentage points. χ^2^ = 6.21, *p* = 0.013.

**Table 4:** Quality of care perceptions by gender (independent-samples *t*-tests).

PCEQQ Dimension	Male Mean (SD)	Female Mean (SD)	Mean Difference (95% CI)	*t*-Value	df	*p*-Value	Cohen’s d	Effect Size Interpretation
Overall Quality	3.85 (0.75)	4.12 (0.68)	0.27 (0.07–0.47)	−2.68	198	0.008 *	0.38	Small-to-medium
Communication	3.65 (0.80)	4.10 (0.72)	0.45 (0.18–0.72)	−3.10	198	0.002 *	0.59	Medium
Symptom Management	3.90 (0.70)	4.05 (0.65)	0.15 (−0.04–0.34)	−1.50	198	0.135	0.22	Small
Emotional Support	3.70 (0.82)	3.95 (0.78)	0.25 (−0.01–0.51)	−1.95	198	0.052	0.31	Small-to-medium

Note: PCEQQ = Palliative Care Experience and Quality Questionnaire. All items were measured on a 5-point Likert scale (1 = very dissatisfied to 5 = very satisfied). Domain scores were calculated as mean of item responses within each domain. CI = confidence interval. * = *p* < 0.05 (statistically significant). Equal variances assumption tested via Levene’s test; assumption met for all comparisons (*p* > 0.05).

**Table 5:** Item-level analysis of PCEQQ quality perceptions by gender.

PCEQQ Item	Male Mean (SD)	Female Mean (SD)	*t*-Value	*p*-Value	Cohen’s d	Significant?
Domain: Communication						
1. Healthcare providers listened to my concerns	3.68 (0.91)	4.15 (0.76)	−3.22	0.002 *	0.53	Yes
2. I received clear information about my condition	3.72 (0.85)	4.18 (0.68)	−3.46	0.001 *	0.57	Yes
3. Healthcare providers understood my feelings	3.58 (0.89)	4.05 (0.79)	−3.38	0.001 *	0.55	Yes
4. Communication was respectful and dignified	3.65 (0.84)	4.12 (0.70)	−3.52	0.001 *	0.58	Yes
5. My questions were answered satisfactorily	3.57 (0.92)	4.02 (0.83)	−3.00	0.003 *	0.50	Yes
Communication Domain Mean	3.65 (0.80)	4.10 (0.72)	−3.10	0.002 *	0.59	Yes
Domain: Symptom Management						
6. My pain was well-controlled	3.92 (0.76)	4.08 (0.68)	−1.44	0.151	0.21	No
7. My shortness of breath was managed	3.85 (0.82)	4.02 (0.72)	−1.38	0.169	0.21	No
8. My nausea/vomiting was controlled	3.95 (0.75)	4.08 (0.65)	−1.18	0.239	0.17	No
9. My constipation was managed	3.88 (0.79)	4.02 (0.71)	−1.22	0.223	0.18	No
10. My fatigue was addressed	3.90 (0.80)	4.05 (0.68)	−1.30	0.195	0.19	No
Symptom Management Domain Mean	3.90 (0.70)	4.05 (0.65)	−1.50	0.135	0.22	No
Domain: Emotional Support						
11. I felt emotionally supported	3.72 (0.88)	3.98 (0.82)	−2.07	0.040 *	0.30	Yes
12. My fears and concerns were discussed	3.65 (0.90)	3.92 (0.84)	−2.04	0.043 *	0.30	Yes
13. Spiritual/existential concerns were addressed	3.68 (0.92)	3.92 (0.86)	−1.80	0.073	0.27	No
14. My family received emotional support	3.75 (0.89)	4.02 (0.81)	−2.08	0.039 *	0.31	Yes
15. My dignity was maintained throughout care	3.70 (0.91)	3.95 (0.85)	−1.88	0.062	0.28	No
Emotional Support Domain Mean	3.70 (0.82)	3.95 (0.78)	−1.95	0.052	0.31	Borderline
Domain: Overall Quality						
16. Overall, I am satisfied with PC services	3.88 (0.79)	4.15 (0.72)	−2.40	0.017 *	0.36	Yes
17. PC improved my quality of life	3.82 (0.82)	4.10 (0.73)	−2.21	0.028 *	0.35	Yes
18. PC addressed my holistic needs	3.85 (0.80)	4.12 (0.71)	−2.35	0.020 *	0.36	Yes
19. Continuity of care was maintained	3.85 (0.78)	4.08 (0.70)	−2.05	0.042 *	0.31	Yes
20. Healthcare providers were attentive to my needs	3.85 (0.81)	4.12 (0.73)	−2.29	0.023 *	0.34	Yes
Overall Quality Domain Mean	3.85 (0.75)	4.12 (0.68)	−2.68	0.008 *	0.38	Yes

Note: PCEQQ = Palliative Care Experience and Quality Questionnaire. All items were measured on a 5-point Likert scale (1 = very dissatisfied to 5 = very satisfied). Domain means were calculated as average of 5 items within each domain. * = *p* < 0.05 (statistically significant). Items in bold rows represent domain summary scores. All communication items (1–5) reached individual significance; symptom management items (6–10) showed no individual significance; emotional support items (11–15) showed mixed results; all overall quality items (16–20) reached individual significance. To facilitate rapid identification of non-significant findings within this dense table, items that did not reach statistical significance (*p* ≥ 0.05) are presented without asterisks; readers seeking only the significant items within each domain are directed to the domain summary rows (bolded) and to items marked with *. Domains are visually separated by the domain mean row to aid quick navigation across the four care dimensions.

**Table 6:** Stratified analysis of quality perceptions by gender and disease stage.

PCEQQ Dimension	Disease Stage	Male Mean (SD)	Female Mean (SD)	Mean Difference	*t*-Value	*p*-Value	Cohen’s d	*n* (M/F)
Overall Quality	Early (I–II)	3.88 (0.73)	4.15 (0.69)	0.27	−1.87	0.065	0.37	45/45
	Advanced (III–IV)	3.83 (0.77)	4.10 (0.66)	0.27	−2.04	0.041	0.36	54/56
Communication	Early (I–II)	3.68 (0.84)	4.15 (0.68)	0.47	−2.65	0.009	0.62	45/45
	Advanced (III–IV)	3.62 (0.76)	4.07 (0.75)	0.45	−2.53	0.008	0.57	54/56
Symptom Management	Early (I–II)	3.92 (0.71)	4.08 (0.63)	0.16	−0.96	0.338	0.24	45/45
	Advanced (III–IV)	3.88 (0.69)	4.02 (0.67)	0.14	−1.08	0.282	0.20	54/56
Emotional Support	Early (I–II)	3.72 (0.84)	3.98 (0.76)	0.26	−1.61	0.111	0.32	45/45
	Advanced (III–IV)	3.68 (0.81)	3.93 (0.80)	0.25	−1.60	0.112	0.31	54/56

Note: Early stage = Stage I–II; Advanced stage = Stage III–IV. Disease stage did not significantly confound gender differences in communication satisfaction (medium effect sizes persisted across both stage groups) or overall quality satisfaction. Symptom management and emotional support showed consistent patterns across stage groups.

**Table 7:** Logistic regression model predicting palliative care access.

Predictor Variable	Unadjusted OR (95% CI)	*p*-Value (Unadjusted)	Adjusted OR (95% CI)	*p*-Value (Adjusted)	95% CI
Female Gender (ref: Male)	1.46 (1.09–2.31)	0.013 *	1.52 (1.10–2.42)	0.009 *	1.10–2.42
Age (per year)	-	-	0.98 (0.96–1.00)	0.612	0.96–1.00
Income ≥ 10,001 SAR (ref: <10,001)	-	-	1.28 (0.85–1.95)	0.089	0.85–1.95
Model Statistics					
Nagelkerke R^2^	-	-	0.084	-	-
−2 Log Likelihood	-	-	254.32	-	-

Note: OR = odds ratio; CI = confidence interval. * = *p* < 0.05 (statistically significant). The adjusted model shows that female gender remains a significant independent predictor of PC access after controlling for age and income. Nagelkerke R^2^ = 0.084 indicates that the model explains approximately 8.4% of the variance in PC access; additional factors not measured in this study are likely to explain the remaining variance.

**Table 8:** Summary of key statistical findings by outcome domain.

Outcome Domain	Finding	Statistic	Effect Size	Interpretation
AWARENESS				
High PC awareness	Females 69.3% vs. Males 47.5%	χ^2^ = 7.23, *p* = 0.008 *	21.8 pp difference	Large practical difference
Odds of high awareness	Female advantage	OR = 2.48, 95% CI: 1.29–4.76, *p* = 0.006 *	-	Females 2.5 × more likely
Understanding PC concept	Females (M = 4.28) vs. Males (M = 3.95)	t = −2.94, *p* = 0.004 *	d = 0.42	Small effect
PC early appropriateness	Females (M = 4.35) vs. Males (M = 3.87)	t = −3.62, *p* < 0.001 *	d = 0.51	Medium effect
ACCESS				
Accessed PC services	Females 64.4% vs. Males 47.5%	χ^2^ = 6.21, *p* = 0.013 *	16.9 pp difference	Moderate practical difference
Odds of PC access (unadjusted)	Female advantage	OR = 1.46, 95% CI: 1.09–2.31, *p* = 0.013 *	-	Females 46% more likely
Odds of PC access (adjusted)	Female advantage after adjustment	OR = 1.52, 95% CI: 1.10–2.42, *p* = 0.009 *	-	Persistent effect; not confounded
QUALITY PERCEPTIONS				
Overall Quality	Females (M = 4.12) vs. Males (M = 3.85)	t = −2.68, *p* = 0.008 *	d = 0.38	Small-to-medium effect
Communication	Females (M = 4.10) vs. Males (M = 3.65)	t = −3.10, *p* = 0.002 *	d = 0.59	Medium effect (LARGEST)
Symptom Management	Females (M = 4.05) vs. Males (M = 3.90)	t = −1.50, *p* = 0.135	d = 0.22	No significant difference
Emotional Support	Females (M = 3.95) vs. Males (M = 3.70)	t = −1.95, *p* = 0.052	d = 0.31	Borderline significance

Note: pp = percentage points. * = *p* < 0.05 (statistically significant). Communication satisfaction demonstrated the most robust and clinically meaningful gender difference (medium effect size, d = 0.59). Overall quality and awareness showed small-to-medium effects. Symptom management was equitable across genders. Emotional support was borderline significant.

## Data Availability

The original contributions presented in this study are included in the article. Further inquiries can be directed to the corresponding author.
